# *N*-Cyclo­pentyl-3-(4-hydr­oxy-6-oxo-1,6-dihydro­pyrimidin-5-yl)-3-*p*-tolyl­propanamide

**DOI:** 10.1107/S1600536809001391

**Published:** 2009-02-06

**Authors:** Xing-Han Wang, Wen-Juan Hao, Shu-Jiang Tu

**Affiliations:** a School of Chemistry and Chemical Engineering, Xuzhou Normal University, Xuzhou 221116, People’s Republic of China

## Abstract

In the mol­ecule of the title compound, C_19_H_23_N_3_O_3_, the six-membered rings are oriented at a dihedral angle of 73.06 (3)°. The cyclo­pentyl ring adopts an envelope conformation. In the crystal structure, inter­molecular N—H⋯O and O—H⋯N hydrogen bonds link the mol­ecules. In the tolyl ring, the H atoms and all but one of the C atoms are disordered over two positions and were refined with occupancies of 0.51 (3) and 0.49 (3).

## Related literature

For general background, see: Johar *et al.* (2005 ▶); Janeba *et al.* (2005 ▶); Soloducho *et al.* (2003 ▶); Mathews & Asokan (2007 ▶); Lagoja (2005 ▶); Michael (2005 ▶); Erian (1993 ▶). For bond-length data, see: Allen *et al.* (1987 ▶).
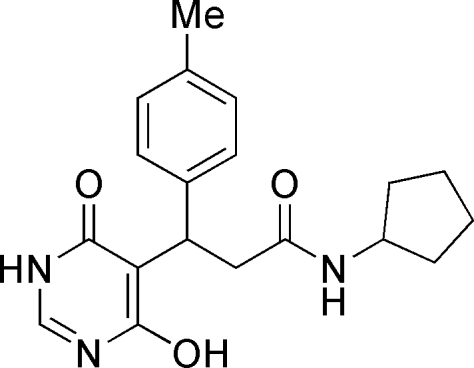


## Experimental

### 

#### Crystal data

C_19_H_23_N_3_O_3_
*M* *_r_* = 341.40
Monoclinic, 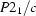

*a* = 11.6798 (10) Å
*b* = 14.8279 (16) Å
*c* = 11.8422 (12) Å
β = 115.022 (2)°
*V* = 1858.4 (3) Å^3^
*Z* = 4
Mo *K*α radiation
μ = 0.08 mm^−1^
*T* = 298 (2) K
0.40 × 0.38 × 0.23 mm

#### Data collection

Bruker SMART CCD area-detector diffractometer
Absorption correction: multi-scan (*SADABS*; Bruker, 1998 ▶) *T*_min_ = 0.967, *T*_max_ = 0.981
9104 measured reflections
3262 independent reflections
1965 reflections with *I* > 2σ(*I*)
*R*_int_ = 0.034

#### Refinement

*R*[*F* ^2^ > 2σ(*F* ^2^)] = 0.041
*wR*(*F* ^2^) = 0.130
*S* = 1.03
3262 reflections
281 parameters
H-atom parameters constrained
Δρ_max_ = 0.17 e Å^−3^
Δρ_min_ = −0.21 e Å^−3^

### 

Data collection: *SMART* (Bruker, 1998 ▶); cell refinement: *SAINT* (Bruker, 1998 ▶); data reduction: *SAINT*; program(s) used to solve structure: *SHELXS97* (Sheldrick, 2008 ▶); program(s) used to refine structure: *SHELXL97* (Sheldrick, 2008 ▶); molecular graphics: *SHELXTL* (Sheldrick, 2008 ▶); software used to prepare material for publication: *SHELXTL*.

## Supplementary Material

Crystal structure: contains datablocks global, I. DOI: 10.1107/S1600536809001391/hk2612sup1.cif

Structure factors: contains datablocks I. DOI: 10.1107/S1600536809001391/hk2612Isup2.hkl

Additional supplementary materials:  crystallographic information; 3D view; checkCIF report

## Figures and Tables

**Table 1:** Hydrogen-bond geometry (Å, °)

*D*—H⋯*A*	*D*—H	H⋯*A*	*D*⋯*A*	*D*—H⋯*A*
N1—H1⋯O3^i^	0.86	1.93	2.692 (3)	147
O2—H2⋯N2^ii^	0.82	1.83	2.639 (3)	167
N3—H3⋯O1^iii^	0.86	2.20	3.017 (3)	158

Symmetry codes: (i) 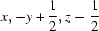; (ii) 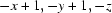; (iii) 
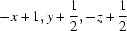
.

**Fig. 1.**
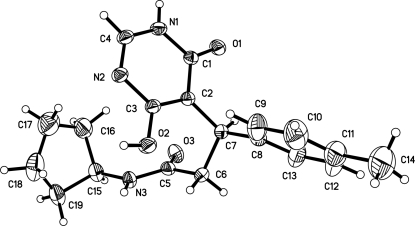
The molecular structure of the title molecule, with the atom-numbering scheme. Displacement ellipsoids are drawn at the 50% probability level.

## supplementary crystallographic information

### Comment

The pyrimidines and their derivatives as a class of extremely important heterocyclic compounds are used in a wide array of synthetic and industrial applications. Not only they are an integral part of the genetic materials, *viz*. DNA and RNA as nucleotides and nucleosides but also play critical roles especially in pharmaceutical fields (Johar *et al.*, 2005; Janeba *et al.*, 2005). Some pyrimidine derivatives can give stable and good quality nanomaterials having many important electrical and optical properties (Soloducho *et al.*, 2003; Mathews & Asokan, 2007), and also used as functional materials (Lagoja, 2005; Michael, 2005; Erian, 1993). We report herein the crystal structure of the title compound.

In the molecule of the title compound (Fig 1), the bond lengths (Allen *et al.*,  1987) and angles are within normal ranges. Rings A (N1/N2/C1-C4) and B (C8-C13) are, of course, planar, and they are oriented at a dihedral angle of 73.06 (3)°. The five-membered ring C (C15-C19) adopts envelope conformation with C15 atom displaced by -0.511 (3) Å from the plane of the other ring atoms.

In the crystal structure, intermolecular N-H···O and O-H···N hydrogen bonds (Table 1) link the molecules, in which they may be effective in the stabilization of the structure.

### Experimental

The title compound was prepared by the reaction of *p*-tolylidene-Meldrum's acid (1 mmol) with 6-hydroxypyrimidin-4(3*H*)-one (1 mmol) and cyclopentanamine (1 mmol) at 373 K in glacial acetic acid under microwave irradiation (maximum power 250 W, initial power 100 W) for 20 min (yield; 87%, m.p. 547–549 K). Crystals suitable for X-ray analysis were obtained from an ethanol solution by slow evaporation.

### Refinement

In the tolyl ring, atoms C9-C14 and H9, H10, H12, H13, H14A, H14B, H14C were disordered over two positions. During the refinement process the disordered atoms were refined with occupancies of 0.51 (3) and 0.49 (3). H atoms were positioned geometrically, with O-H = 0.82 Å (for OH), N-H = 0.86 Å (for NH) and C-H = 0.93, 0.98, 0.97 and 0.96 Å for aromatic, methine, methylene and methyl H, respectively, and constrained to ride on their parent atoms, with U_iso_(H) = xU_eq_(C,N,O), where x = 1.5 for methyl and OH H and x = 1.2 for all other H atoms.

### Crystal data

**Table:**

C_19_H_23_N_3_O_3_	*F*(000) = 728
*M**_r_* = 341.40	*D*_x_ = 1.220 Mg m^−^^3^
Monoclinic, *P*2_1_/*c*	Melting point = 547–549 K
Hall symbol: -P 2ybc	Mo *K*α radiation, λ = 0.71073 Å
*a* = 11.6798 (10) Å	Cell parameters from 2142 reflections
*b* = 14.8279 (16) Å	θ = 2.4–25.3°
*c* = 11.8422 (12) Å	µ = 0.08 mm^−^^1^
β = 115.022 (2)°	*T* = 298 K
*V* = 1858.4 (3) Å^3^	Block, colorless
*Z* = 4	0.40 × 0.38 × 0.23 mm

### Data collection

**Table:**

Bruker SMART CCD area-detector diffractometer	3262 independent reflections
Radiation source: fine-focus sealed tube	1965 reflections with *I* > 2σ(*I*)
graphite	*R*_int_ = 0.034
φ and ω scans	θ_max_ = 25.0°, θ_min_ = 1.9°
Absorption correction: multi-scan (*SADABS*; Bruker, 1998)	*h* = −13→13
*T*_min_ = 0.967, *T*_max_ = 0.981	*k* = −17→9
9104 measured reflections	*l* = −14→14

### Refinement

**Table:**

Refinement on *F*^2^	Primary atom site location: structure-invariant direct methods
Least-squares matrix: full	Secondary atom site location: difference Fourier map
*R*[*F*^2^ > 2σ(*F*^2^)] = 0.041	Hydrogen site location: inferred from neighbouring sites
*wR*(*F*^2^) = 0.130	H-atom parameters constrained
*S* = 1.03	*w* = 1/[σ^2^(*F*_o_^2^) + (0.0557*P*)^2^ + 0.3901*P*] where *P* = (*F*_o_^2^ + 2*F*_c_^2^)/3
3262 reflections	(Δ/σ)_max_ = 0.001
281 parameters	Δρ_max_ = 0.17 e Å^−^^3^
0 restraints	Δρ_min_ = −0.21 e Å^−^^3^

### Special details

**Table:**

Geometry. All e.s.d.'s (except the e.s.d. in the dihedral angle between two l.s. planes) are estimated using the full covariance matrix. The cell e.s.d.'s are taken into account individually in the estimation of e.s.d.'s in distances, angles and torsion angles; correlations between e.s.d.'s in cell parameters are only used when they are defined by crystal symmetry. An approximate (isotropic) treatment of cell e.s.d.'s is used for estimating e.s.d.'s involving l.s. planes.
Refinement. Refinement of *F*^2^ against ALL reflections. The weighted *R*-factor *wR* and goodness of fit *S* are based on *F*^2^, conventional *R*-factors *R* are based on *F*, with *F* set to zero for negative *F*^2^. The threshold expression of *F*^2^ > σ(*F*^2^) is used only for calculating *R*-factors(gt) *etc*. and is not relevant to the choice of reflections for refinement. *R*-factors based on *F*^2^ are statistically about twice as large as those based on *F*, and *R*- factors based on ALL data will be even larger.

### Fractional atomic coordinates and isotropic or equivalent isotropic displacement parameters (Å^2^)

**Table:**

	*x*	*y*	*z*	*U*_iso_*/*U*_eq_	Occ. (<1)
O1	0.51033 (15)	0.18524 (10)	0.24325 (14)	0.0478 (4)	
O2	0.58111 (16)	0.48802 (10)	0.17050 (14)	0.0519 (5)	
H2	0.5650	0.5192	0.1084	0.078*	
O3	0.36199 (16)	0.38438 (11)	0.37082 (15)	0.0565 (5)	
N1	0.45054 (17)	0.24273 (12)	0.04951 (16)	0.0415 (5)	
H1	0.4223	0.1907	0.0181	0.050*	
N2	0.47888 (17)	0.39142 (12)	0.00944 (16)	0.0407 (5)	
N3	0.36553 (17)	0.50839 (13)	0.26475 (16)	0.0436 (5)	
H3	0.4105	0.5496	0.2520	0.052*	
C1	0.5061 (2)	0.25152 (15)	0.1789 (2)	0.0366 (5)	
C2	0.5537 (2)	0.33982 (14)	0.22258 (19)	0.0348 (5)	
C3	0.5389 (2)	0.40604 (15)	0.13651 (19)	0.0369 (5)	
C4	0.4392 (2)	0.31040 (16)	−0.0272 (2)	0.0430 (6)	
H4	0.4004	0.2992	−0.1125	0.052*	
C5	0.4234 (2)	0.44262 (15)	0.3448 (2)	0.0414 (6)	
C6	0.5653 (2)	0.44127 (15)	0.4011 (2)	0.0433 (6)	
H6A	0.5977	0.4429	0.4912	0.052*	
H6B	0.5957	0.4945	0.3746	0.052*	
C7	0.6153 (2)	0.35575 (14)	0.36151 (19)	0.0385 (5)	
H7	0.5905	0.3047	0.3988	0.046*	
C8	0.7589 (2)	0.35570 (16)	0.4198 (2)	0.0493 (6)	
C9	0.822 (3)	0.3612 (18)	0.342 (3)	0.059 (4)	0.51 (3)
H9	0.7759	0.3626	0.2559	0.071*	0.51 (3)
C9'	0.838 (3)	0.382 (2)	0.370 (3)	0.069 (5)	0.49 (3)
H9'	0.8043	0.4040	0.2884	0.082*	0.49 (3)
C10	0.952 (3)	0.3646 (19)	0.392 (3)	0.074 (4)	0.51 (3)
H10	0.9918	0.3698	0.3383	0.089*	0.51 (3)
C10'	0.970 (3)	0.377 (2)	0.437 (2)	0.083 (6)	0.49 (3)
H10'	1.0222	0.3923	0.3991	0.100*	0.49 (3)
C11	1.025 (4)	0.361 (2)	0.517 (3)	0.072 (6)	0.51 (3)
C11'	1.023 (4)	0.350 (3)	0.561 (3)	0.080 (8)	0.49 (3)
C12	0.964 (2)	0.3535 (19)	0.596 (2)	0.082 (5)	0.51 (3)
H12	1.0112	0.3506	0.6818	0.098*	0.51 (3)
C12'	0.943 (3)	0.3240 (16)	0.611 (2)	0.084 (5)	0.49 (3)
H12'	0.9764	0.3037	0.6925	0.101*	0.49 (3)
C13	0.833 (4)	0.351 (2)	0.545 (4)	0.070 (6)	0.51 (3)
H13	0.7942	0.3453	0.5991	0.084*	0.51 (3)
C13'	0.813 (4)	0.327 (2)	0.544 (4)	0.070 (5)	0.49 (3)
H13'	0.7611	0.3088	0.5821	0.084*	0.49 (3)
C14	1.1676 (18)	0.3628 (15)	0.573 (2)	0.110 (5)	0.51 (3)
H14A	1.1995	0.3023	0.5824	0.164*	0.51 (3)
H14B	1.1991	0.3914	0.6534	0.164*	0.51 (3)
H14C	1.1948	0.3960	0.5194	0.164*	0.51 (3)
C14'	1.1691 (19)	0.3465 (18)	0.635 (2)	0.121 (7)	0.49 (3)
H14D	1.1905	0.3341	0.7209	0.181*	0.49 (3)
H14E	1.2045	0.4035	0.6277	0.181*	0.49 (3)
H14F	1.2024	0.2998	0.6011	0.181*	0.49 (3)
C15	0.2281 (2)	0.51310 (17)	0.1977 (2)	0.0539 (7)	
H15	0.1881	0.5034	0.2546	0.065*	
C16	0.1793 (3)	0.4455 (2)	0.0910 (3)	0.0839 (10)	
H16A	0.2334	0.3927	0.1108	0.101*	
H16B	0.0941	0.4264	0.0743	0.101*	
C17	0.1810 (3)	0.4946 (3)	−0.0198 (3)	0.1004 (12)	
H17A	0.2484	0.4714	−0.0390	0.121*	
H17B	0.1014	0.4860	−0.0921	0.121*	
C18	0.2015 (3)	0.5919 (3)	0.0119 (3)	0.0961 (12)	
H18A	0.1392	0.6282	−0.0534	0.115*	
H18B	0.2851	0.6103	0.0221	0.115*	
C19	0.1885 (3)	0.60299 (19)	0.1318 (3)	0.0689 (8)	
H19A	0.1018	0.6169	0.1158	0.083*	
H19B	0.2428	0.6510	0.1816	0.083*	

### Atomic displacement parameters (Å^2^)

**Table:**

	*U*^11^	*U*^22^	*U*^33^	*U*^12^	*U*^13^	*U*^23^
N1	0.0550 (12)	0.0301 (10)	0.0396 (11)	−0.0049 (9)	0.0201 (9)	−0.0058 (9)
N2	0.0532 (12)	0.0373 (11)	0.0322 (10)	−0.0031 (9)	0.0187 (9)	−0.0019 (8)
N3	0.0434 (12)	0.0401 (11)	0.0451 (11)	0.0029 (9)	0.0167 (9)	0.0092 (9)
O1	0.0608 (11)	0.0341 (9)	0.0479 (10)	0.0002 (8)	0.0222 (8)	0.0041 (7)
O2	0.0760 (12)	0.0353 (9)	0.0387 (9)	−0.0096 (8)	0.0189 (8)	0.0005 (7)
O3	0.0634 (12)	0.0471 (10)	0.0624 (11)	0.0063 (9)	0.0300 (9)	0.0180 (9)
C1	0.0384 (13)	0.0360 (13)	0.0370 (13)	0.0040 (10)	0.0176 (10)	−0.0014 (10)
C2	0.0396 (13)	0.0326 (12)	0.0320 (12)	0.0015 (10)	0.0150 (10)	−0.0005 (10)
C3	0.0435 (13)	0.0342 (13)	0.0324 (13)	−0.0015 (10)	0.0155 (11)	−0.0039 (10)
C4	0.0534 (15)	0.0426 (15)	0.0342 (13)	−0.0021 (12)	0.0196 (11)	−0.0062 (11)
C5	0.0538 (15)	0.0362 (13)	0.0349 (13)	0.0062 (12)	0.0195 (11)	−0.0003 (11)
C6	0.0509 (15)	0.0420 (14)	0.0310 (12)	0.0038 (11)	0.0114 (11)	−0.0028 (10)
C7	0.0445 (14)	0.0340 (12)	0.0323 (12)	0.0025 (10)	0.0116 (10)	0.0012 (10)
C8	0.0484 (15)	0.0392 (14)	0.0495 (16)	0.0021 (12)	0.0102 (13)	−0.0047 (12)
C9	0.041 (8)	0.064 (9)	0.064 (9)	−0.007 (6)	0.014 (7)	−0.012 (6)
C9'	0.049 (7)	0.076 (14)	0.062 (11)	−0.009 (9)	0.007 (8)	0.001 (7)
C10	0.047 (9)	0.092 (9)	0.070 (13)	−0.004 (6)	0.013 (10)	−0.010 (8)
C10'	0.047 (10)	0.108 (12)	0.074 (19)	−0.011 (7)	0.006 (12)	−0.012 (11)
C11	0.050 (8)	0.084 (9)	0.067 (18)	−0.003 (6)	0.009 (12)	0.005 (13)
C11'	0.054 (10)	0.090 (13)	0.071 (18)	0.006 (10)	0.003 (12)	−0.003 (12)
C12	0.054 (10)	0.097 (14)	0.068 (9)	−0.011 (10)	0.000 (7)	−0.004 (8)
C12'	0.058 (10)	0.081 (11)	0.078 (7)	−0.006 (7)	−0.007 (6)	0.014 (7)
C13	0.054 (12)	0.078 (15)	0.057 (6)	−0.006 (9)	0.003 (7)	−0.004 (10)
C13'	0.054 (12)	0.078 (15)	0.058 (6)	−0.006 (9)	0.003 (7)	−0.003 (10)
C14	0.056 (5)	0.126 (9)	0.106 (13)	0.000 (5)	−0.005 (9)	0.007 (10)
C14'	0.054 (5)	0.150 (14)	0.112 (13)	0.000 (7)	−0.010 (10)	−0.010 (12)
C15	0.0443 (15)	0.0508 (16)	0.0633 (17)	0.0038 (12)	0.0195 (13)	0.0137 (13)
C16	0.0547 (19)	0.068 (2)	0.097 (3)	−0.0028 (16)	0.0014 (17)	−0.0018 (19)
C17	0.084 (3)	0.133 (4)	0.082 (3)	0.023 (2)	0.033 (2)	−0.003 (2)
C18	0.077 (2)	0.119 (3)	0.078 (2)	−0.004 (2)	0.0194 (19)	0.036 (2)
C19	0.0533 (17)	0.0587 (18)	0.083 (2)	0.0101 (14)	0.0174 (15)	0.0209 (16)

### Geometric parameters (Å, °)

**Table:**

O1—C1	1.232 (2)	C12—C13	1.39 (5)
O2—C3	1.310 (2)	C12—H12	0.9300
O2—H2	0.8200	C13—H13	0.9300
O3—C5	1.242 (3)	C14—H14A	0.9600
N1—C4	1.322 (3)	C14—H14B	0.9600
N1—C1	1.395 (3)	C14—H14C	0.9600
N1—H1	0.8600	C9'—C10'	1.41 (5)
N2—C4	1.295 (3)	C9'—H9'	0.9300
N2—C3	1.382 (3)	C10'—C11'	1.38 (3)
N3—C5	1.328 (3)	C10'—H10'	0.9300
N3—C15	1.460 (3)	C11'—C12'	1.35 (4)
N3—H3	0.8600	C11'—C14'	1.56 (4)
C1—C2	1.431 (3)	C12'—C13'	1.38 (6)
C2—C3	1.373 (3)	C12'—H12'	0.9300
C2—C7	1.510 (3)	C13'—H13'	0.9300
C4—H4	0.9300	C14'—H14D	0.9600
C5—C6	1.503 (3)	C14'—H14E	0.9600
C6—C7	1.549 (3)	C14'—H14F	0.9600
C6—H6A	0.9700	C15—C19	1.515 (3)
C6—H6B	0.9700	C15—C16	1.523 (4)
C7—C8	1.520 (3)	C15—H15	0.9800
C7—H7	0.9800	C16—C17	1.509 (5)
C8—C9'	1.35 (4)	C16—H16A	0.9700
C8—C13	1.37 (4)	C16—H16B	0.9700
C8—C13'	1.40 (4)	C17—C18	1.485 (5)
C8—C9	1.41 (3)	C17—H17A	0.9700
C9—C10	1.38 (5)	C17—H17B	0.9700
C9—H9	0.9300	C18—C19	1.500 (4)
C10—C11	1.37 (3)	C18—H18A	0.9700
C10—H10	0.9300	C18—H18B	0.9700
C11—C12	1.39 (4)	C19—H19A	0.9700
C11—C14	1.51 (4)	C19—H19B	0.9700
			
C3—O2—H2	109.5	C11—C14—H14A	109.5
C4—N1—C1	123.31 (19)	C11—C14—H14B	109.5
C4—N1—H1	118.3	H14A—C14—H14B	109.5
C1—N1—H1	118.3	C11—C14—H14C	109.5
C4—N2—C3	117.10 (19)	H14A—C14—H14C	109.5
C5—N3—C15	122.2 (2)	H14B—C14—H14C	109.5
C5—N3—H3	118.9	C8—C9'—C10'	121 (3)
C15—N3—H3	118.9	C8—C9'—H9'	119.3
O1—C1—N1	118.9 (2)	C10'—C9'—H9'	119.3
O1—C1—C2	126.8 (2)	C11'—C10'—C9'	121 (3)
N1—C1—C2	114.31 (19)	C11'—C10'—H10'	119.7
C3—C2—C1	118.59 (19)	C9'—C10'—H10'	119.7
C3—C2—C7	123.34 (18)	C12'—C11'—C10'	118 (3)
C1—C2—C7	118.06 (18)	C12'—C11'—C14'	123 (2)
O2—C3—C2	121.54 (19)	C10'—C11'—C14'	120 (2)
O2—C3—N2	115.58 (18)	C11'—C12'—C13'	123 (3)
C2—C3—N2	122.88 (19)	C11'—C12'—H12'	118.7
N2—C4—N1	123.8 (2)	C13'—C12'—H12'	118.7
N2—C4—H4	118.1	C12'—C13'—C8	120 (3)
N1—C4—H4	118.1	C12'—C13'—H13'	119.9
O3—C5—N3	121.0 (2)	C8—C13'—H13'	119.9
O3—C5—C6	121.7 (2)	C11'—C14'—H14D	109.5
N3—C5—C6	117.3 (2)	C11'—C14'—H14E	109.5
C5—C6—C7	111.29 (18)	H14D—C14'—H14E	109.5
C5—C6—H6A	109.4	C11'—C14'—H14F	109.5
C7—C6—H6A	109.4	H14D—C14'—H14F	109.5
C5—C6—H6B	109.4	H14E—C14'—H14F	109.5
C7—C6—H6B	109.4	N3—C15—C19	110.4 (2)
H6A—C6—H6B	108.0	N3—C15—C16	111.1 (2)
C2—C7—C8	114.88 (19)	C19—C15—C16	103.1 (2)
C2—C7—C6	112.99 (17)	N3—C15—H15	110.7
C8—C7—C6	110.36 (18)	C19—C15—H15	110.7
C2—C7—H7	105.9	C16—C15—H15	110.7
C8—C7—H7	105.9	C17—C16—C15	105.4 (3)
C6—C7—H7	105.9	C17—C16—H16A	110.7
C9'—C8—C13	104.8 (18)	C15—C16—H16A	110.7
C9'—C8—C13'	118 (2)	C17—C16—H16B	110.7
C13—C8—C9	116 (2)	C15—C16—H16B	110.7
C13'—C8—C9	125 (2)	H16A—C16—H16B	108.8
C9'—C8—C7	129.0 (14)	C18—C17—C16	108.0 (3)
C13—C8—C7	124.3 (17)	C18—C17—H17A	110.1
C13'—C8—C7	113.3 (17)	C16—C17—H17A	110.1
C9—C8—C7	119.2 (12)	C18—C17—H17B	110.1
C10—C9—C8	121 (2)	C16—C17—H17B	110.1
C10—C9—H9	119.6	H17A—C17—H17B	108.4
C8—C9—H9	119.6	C17—C18—C19	106.0 (3)
C11—C10—C9	122 (3)	C17—C18—H18A	110.5
C11—C10—H10	119.0	C19—C18—H18A	110.5
C9—C10—H10	119.0	C17—C18—H18B	110.5
C10—C11—C12	118 (3)	C19—C18—H18B	110.5
C10—C11—C14	123 (3)	H18A—C18—H18B	108.7
C12—C11—C14	119 (2)	C18—C19—C15	105.1 (3)
C13—C12—C11	120 (3)	C18—C19—H19A	110.7
C13—C12—H12	120.1	C15—C19—H19A	110.7
C11—C12—H12	120.1	C18—C19—H19B	110.7
C8—C13—C12	123 (3)	C15—C19—H19B	110.7
C8—C13—H13	118.5	H19A—C19—H19B	108.8
C12—C13—H13	118.5		
			
C4—N1—C1—O1	178.5 (2)	C7—C8—C9—C10	177.6 (19)
C4—N1—C1—C2	−1.8 (3)	C8—C9—C10—C11	2(4)
O1—C1—C2—C3	−179.2 (2)	C9—C10—C11—C12	0(5)
N1—C1—C2—C3	1.2 (3)	C9—C10—C11—C14	179 (3)
O1—C1—C2—C7	−0.4 (3)	C10—C11—C12—C13	0(5)
N1—C1—C2—C7	179.93 (18)	C14—C11—C12—C13	−180 (3)
C1—C2—C3—O2	−179.52 (19)	C9'—C8—C13—C12	−13 (3)
C7—C2—C3—O2	1.8 (3)	C13'—C8—C13—C12	126 (14)
C1—C2—C3—N2	0.7 (3)	C9—C8—C13—C12	2(4)
C7—C2—C3—N2	−178.0 (2)	C7—C8—C13—C12	−178 (2)
C4—N2—C3—O2	178.1 (2)	C11—C12—C13—C8	−1(5)
C4—N2—C3—C2	−2.1 (3)	C13—C8—C9'—C10'	16 (3)
C3—N2—C4—N1	1.5 (3)	C13'—C8—C9'—C10'	3(3)
C1—N1—C4—N2	0.5 (3)	C9—C8—C9'—C10'	−118 (10)
C15—N3—C5—O3	−3.8 (3)	C7—C8—C9'—C10'	−179.8 (17)
C15—N3—C5—C6	175.70 (19)	C8—C9'—C10'—C11'	−4(5)
O3—C5—C6—C7	63.5 (3)	C9'—C10'—C11'—C12'	4(5)
N3—C5—C6—C7	−115.9 (2)	C9'—C10'—C11'—C14'	−179 (2)
C3—C2—C7—C8	−80.4 (3)	C10'—C11'—C12'—C13'	−2(5)
C1—C2—C7—C8	100.9 (2)	C14'—C11'—C12'—C13'	−179 (3)
C3—C2—C7—C6	47.4 (3)	C11'—C12'—C13'—C8	1(5)
C1—C2—C7—C6	−131.3 (2)	C9'—C8—C13'—C12'	−1(4)
C5—C6—C7—C2	50.0 (2)	C13—C8—C13'—C12'	−47 (10)
C5—C6—C7—C8	−179.86 (19)	C9—C8—C13'—C12'	18 (4)
C2—C7—C8—C9'	31.9 (16)	C7—C8—C13'—C12'	−179 (2)
C6—C7—C8—C9'	−97.3 (16)	C5—N3—C15—C19	169.8 (2)
C2—C7—C8—C13	−166.3 (17)	C5—N3—C15—C16	−76.5 (3)
C6—C7—C8—C13	64.5 (18)	N3—C15—C16—C17	−89.0 (3)
C2—C7—C8—C13'	−150.5 (16)	C19—C15—C16—C17	29.2 (3)
C6—C7—C8—C13'	80.4 (16)	C15—C16—C17—C18	−12.2 (4)
C2—C7—C8—C9	13.8 (13)	C16—C17—C18—C19	−10.0 (4)
C6—C7—C8—C9	−115.4 (13)	C17—C18—C19—C15	28.6 (3)
C9'—C8—C9—C10	50 (8)	N3—C15—C19—C18	83.0 (3)
C13—C8—C9—C10	−2(3)	C16—C15—C19—C18	−35.7 (3)
C13'—C8—C9—C10	−20 (3)		

### Hydrogen-bond geometry (Å, °)

**Table:**

*D*—H···*A*	*D*—H	H···*A*	*D*···*A*	*D*—H···*A*
N1—H1···O3^i^	0.86	1.93	2.692 (3)	147
O2—H2···N2^ii^	0.82	1.83	2.639 (3)	167
N3—H3···O1^iii^	0.86	2.20	3.017 (3)	158

Symmetry codes: (i) *x*, −*y*+1/2, *z*−1/2; (ii) −*x*+1, −*y*+1, −*z*; (iii) −*x*+1, *y*+1/2, −*z*+1/2.
